# Treatment of Patients with Advanced Biliary Tract Cancer with Either Oxaliplatin, Gemcitabine, and Capecitabine or Cisplatin and Gemcitabine—A Randomized Phase II Trial

**DOI:** 10.3390/cancers12071975

**Published:** 2020-07-20

**Authors:** Alice Markussen, Lars Henrik Jensen, Laura Vittrup Diness, Finn Ole Larsen

**Affiliations:** 1 Department of Oncology, Copenhagen University Hospital, Herlev and Gentofte, 2730 Herlev, Denmark; laura.vittrup.diness@regionh.dk (L.V.D.); finn.ole.larsen@regionh.dk (F.O.L.); 2 Department of Oncology, Vejle University Hospital, 7100 Vejle, Denmark; Lars.Henrik.Jensen@rsyd.dk

**Keywords:** biliary tract cancer, cholangiocarcinoma, randomized phase II trial, chemotherapy, cisplatin, oxaliplatin

## Abstract

This study is an investigator-initiated randomized phase II trial focusing on the treatment of advanced biliary tract cancer with either oxaliplatin 50 mg/m^2^ and gemcitabine 1000 mg/m^2^ on day 1 in a two-week cycle with capecitabine 650 mg/m^2^ twice-daily continuously or cisplatin 25 mg/m^2^ and gemcitabine 1000 mg/m^2^ on day 1 and day 8 in a three-week cycle. One-hundred patients were included. Forty-seven patients received oxaliplatin, gemcitabine, and capecitabine with a median progression-free survival (mPFS) of 5.7 months (95% CI 3.0–7.8) and a median overall survival (mOS) of 8.7 months (95% CI 6.5–11.2). Forty-nine patients received cisplatin and gemcitabine with a mPFS of 7.3 months (95% CI 6.0–8.7) and a mOS of 12.0 months (95% CI 8.3–16.7). This trial confirms a mOS of 12 months with cisplatin and gemcitabine, as found in earlier trials. With a superior tumor control rate of 79% vs. 60% (*p* = 0.045), a difference in the mPFS of 1.6 months (HR = 0.721, *p* = 0.1), and a difference in the mOS of 3.3 months (HR = 0.731, *p* = 0.1), cisplatin and gemcitabine should still be considered the standard first-line treatment for advanced biliary tract cancer.

## 1. Introduction

Biliary tract cancer (BTC) includes cancers arising from the biliary epithelium in the liver, the main ducts of the hilum, and extending into the gallbladder and bile duct. In the Western world, BTC is a rare cancer. It accounts for less than 1% of new cancer diagnoses in the United States, with approximately 12,000 new cases a year [1]. The number of newly registered cases in Denmark is 210 per year in a population of 5.8 million [2]. The only curative treatments are resection or a liver transplant [3]. Unfortunately, most patients have advanced disease when diagnosed, and their treatment possibilities are only palliative [4]. Patients who undergo curative surgery have a high recurrence rate, resulting in a five-year overall survival (OS) rate of 15% for the entire patient population [2].

Before 2010, there was no established standard treatment for advanced BTC [5], though several different approaches have been explored. The main tendencies in these treatments were summarized by Valle et al. [6]. They found that most of the chemotherapy regimens used 5-fluorouracil (5-FU) either alone, with response rates of 25%–32%, or in combination with cisplatin, doxorubicin, epirubicin, hydroxyurea, or methotrexate, where only small improvements were observed compared to 5-FU alone. Other treatments included capecitabine, docetaxel, or gemcitabine. The median overall survival (mOS) in these studies varied from 5.7 to 11+ months [6].

The ABC-01/02 trial [6,7] studied the addition of cisplatin to a gemcitabine-based regimen. ABC-02 showed an increased mOS of 11.7 months with cisplatin and gemcitabine, compared to a mOS of 8.1 months with gemcitabine alone. The median progression-free survival (mPFS) increased from 5.0 to 8.0 months when cisplatin was also used. Even though this regimen is now considered the standard treatment, modifications and substitutions have been tried in the pursuit of a better response, less toxicity, and less time-consuming treatment.

As oxaliplatin is easier to administer than cisplatin, some centers have substituted cisplatin with oxaliplatin. Fiteni et al. [8] performed a systematic review in which they assessed the median of the mOS and a weighted mOS based on 33 studies where patients with advanced BTC were treated with gemcitabine in combination with either oxaliplatin or cisplatin. The authors found that the treatments were comparable with regards to the mOS, but there was lower toxicity in the oxaliplatin group. No randomized trials comparing the two treatments have been conducted.

In the treatment of BTC before 2010, 5-FU had a leading role, often in combination with gemcitabine [9,10], and it is now recommended in the adjuvant setting in the form of capecitabine [11]. In the metastatic setting, a trial that compared capecitabine and oxaliplatin with gemcitabine and oxaliplatin found them to be equally effective [12]. A triple combination using oxaliplatin, gemcitabine, and capecitabine has been tested in several trials [13,14,15,16]. This combination was found to be well-tolerated and with response rates, progression-free survival (PFS), and OS similar to those for cisplatin and gemcitabine.

We were therefore interested in further investigating the treatment of BTC with either oxaliplatin, gemcitabine, and capecitabine or cisplatin and gemcitabine with regards to efficacy and toxicity in a randomized trial.

## 2. Patients and Methods

### 2.1. Study Design

This study is an investigator-initiated randomized phase II study of either oxaliplatin, gemcitabine, and capecitabine or cisplatin and gemcitabine. The inclusion period started in July 2014 and ended in November 2017. During that period, patients with BTC were treated at two centers in Denmark, and both centers participated in the study. Patients with advanced BTC were randomized 1:1 between the two regimens. The randomization was done by permutated block randomization, stratified by performance status. The treatment arm was not blinded to either the patients or the hospital personnel. The primary endpoint was PFS. The secondary endpoints were OS, response rate, and toxicity.

Due to the rarity of this disease, we included 100 patients at the two centers. This did not allow for sufficient power to permit a formal statistical comparison between the two treatment arms. However, for an initial assessment of the two regimens, we were interested in finding out whether a randomized phase III noninferiority trial in an international collaboration would be relevant, feasible, and ethically sound.

All patients provided written informed consent, and the study was carried out in accordance with the Good Clinical Practice guidelines and the provisions of the Declaration of Helsinki.

The trial was approved by the Regional Scientific Ethics Committee VEK no. H-2-2014-026, the Danish Medicine Agency EudraCT no. 2013-004854-46, and was registered in the EU clinical trials register.

### 2.2. Patients

Patients were eligible for the study if they were ≥18 years and had a histopathological diagnosis of nonresectable, recurrent, or metastatic BTC or a cytologic diagnosis of carcinoma, in combination with radiological findings confirming the diagnosis. Intrahepatic, perihilar, extrahepatic, and gallbladder cancers could be included—but not ampullary cancer. All patients with the locally advanced disease were evaluated at a multidisciplinary team conference for resectability, with the participation of hepatobiliary surgeons. Patients had to have an Eastern Cooperative Oncology Group performance status ≤1, blood samples with neutrophil granulocytes ≥1.5 × 10^9^/L, thrombocytes ≥75 × 10^9^/L, bilirubin ≤2× the upper normal limit, and creatinine clearance ≥45 mL/min.

The exclusion criteria were clinically significant comorbidity, concurrent cancer, pregnancy, breastfeeding, or known intolerance to one or more of the study drugs.

### 2.3. Treatments

The experimental arm consisted of oxaliplatin 50 mg/m^2^ every second week, with an infusion time of 30 min, gemcitabine 1000 mg/m^2^ every second week, with an infusion time of 30 min, and capecitabine 650 mg/m^2^ twice-daily and continually in a 2-week cycle. The combined treatment time in the outpatient clinic was 2 h.

The schedule in the experimental arm was chosen based on three previous studies. Firstly, a phase I–II dose-escalating study of oxaliplatin, gemcitabine, and capecitabine [15]. In this study, 56% of the patients developed grade 3 or grade 4 neutropenia when treated in a 2-week cycle with doses of gemcitabine at 1000 mg/m^2^ every second week, oxaliplatin 60 mg/m^2^ every second week, and capecitabine 1000 mg/m^2^ in one week followed by a one-week break. Secondly, on a phase II trial investigating the addition of cetuximab to a regimen consisting of gemcitabine at 1000 mg/m^2^ every second week, a lower dose of oxaliplatin of 50 mg/m^2^ every second week, and capecitabine 650 mg/m^2^ twice-daily [16]. In this study, the treatment was found to be well-tolerated, with a mOS of 12.8 months. Finally, on a retrospective study evaluating the three-drug combination in 194 patients with a mOS of 10.8 months [14]. This study included 33 patients with a performance status of 2 and with a notably shorter survival, which was expected to influence the mOS.

Continuous versus intermittent treatments with capecitabine have previously been investigated, without a significant difference in efficacy or toxicity [17]. As capecitabine is often administered as 1000 mg/m^2^ twice-daily in a 2-weeks-on/1-week-off schedule, an equivalent dose intensity in a continuous treatment would be capecitabine of 650 mg/m^2^ twice-daily. In the phase II trials, a difference in oxaliplatin from 60 to 50 mg seemed to reduced neutropenia; we therefore chose the lower dose to reduce the risk of treatment delays and complications.

In the treatment arm consisting of cisplatin and gemcitabine, each cycle lasted 3 weeks and comprised cisplatin 25 mg/m^2^, with an infusion time of 60 min, and gemcitabine 1000 mg/m^2^, with an infusion time of 30 min, on day 1 and day 8. The combined treatment time in the outpatient clinic was 4 h and 30 min. This schedule was chosen due to a previous phase III trial [7] and regarded as the standard treatment.

Patients were checked before every new cycle, with complete blood counts and an assessment of the liver and renal functions. They were evaluated for symptoms, adverse events, and an estimation of performance status. Dose modifications for each regimen were carried out, if indicated, by predefined criteria.

The treatment was continued until progression, unmanageable toxicity, or a withdrawal of consent.

### 2.4. Assessments

Investigators graded adverse events and toxic effects according to the National Cancer Institute’s Common Toxicity Criteria version 3.0. before each treatment. Tumor response was evaluated by means of CT scans every 12 weeks, measured by the Response Evaluation Criteria in Solid Tumors (RECIST) version 1.1 [18]. The 12-week interval was chosen to mimic the ABC-02 trial. Tumor control was defined as complete response (CR), partial response (PR), or stable disease (SD). Progression was defined as tumor progression according to RECIST 1.1; if the patients’ general condition did not allow for further treatment, or if none of the above, then patients were censored at time of death or last seen.

### 2.5. Statistical Analysis

All analyses were performed using R version 3.5.1 and performed on patients receiving at least one treatment. This excluded four patients. PFS was calculated from the date of first treatment to the date of progression or death from any cause. OS was calculated from the date of first treatment to the date of death from any cause. PFS and OS were analyzed with the use of the Kaplan–Meier curves and likelihood ratio tests on univariate Cox proportional-hazards models. Confidence intervals of the medians were estimates using the Rothman method.

Toxicity was reported as any grade and subdivided as grades 3 and 4. In order to identify statistically significant differences in toxicity, Fisher’s exact test was performed on grade 3 and 4 toxicities.

Differences in patients’ characteristics were tested using Fisher’s exact test, except for age, where a *t*-test was used to test if the populations were significantly different.

Dose intensity (DI) is the amount of drug delivered per unit of time expressed as mg/m^2^/week [19]. Relative DI is the amount of dose delivered compared to what was intended, as defined by the protocol. We calculated the relative DI of cisplatin and oxaliplatin for all 96 patients that received at least one treatment. It was calculated per patient, per cycle, and the mean DI per cycle was reported. In order to compare the relative DI of the two treatments, it was plotted over time rather than by cycle. For the calculations of DI, all patients were censored at the time of treatment discontinuation or after 8 and 12 cycles, depending on the regimen (equivalent to 24 weeks of treatment).

## 3. Results

**Figure 1:**
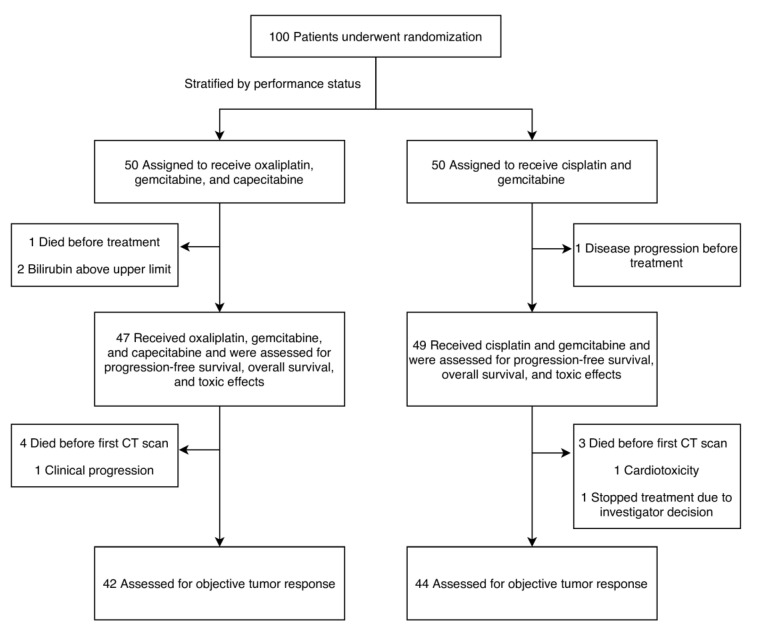
Patient enrollment, randomization, and treatment. CT—computerized tomography.

**Figure 2:**
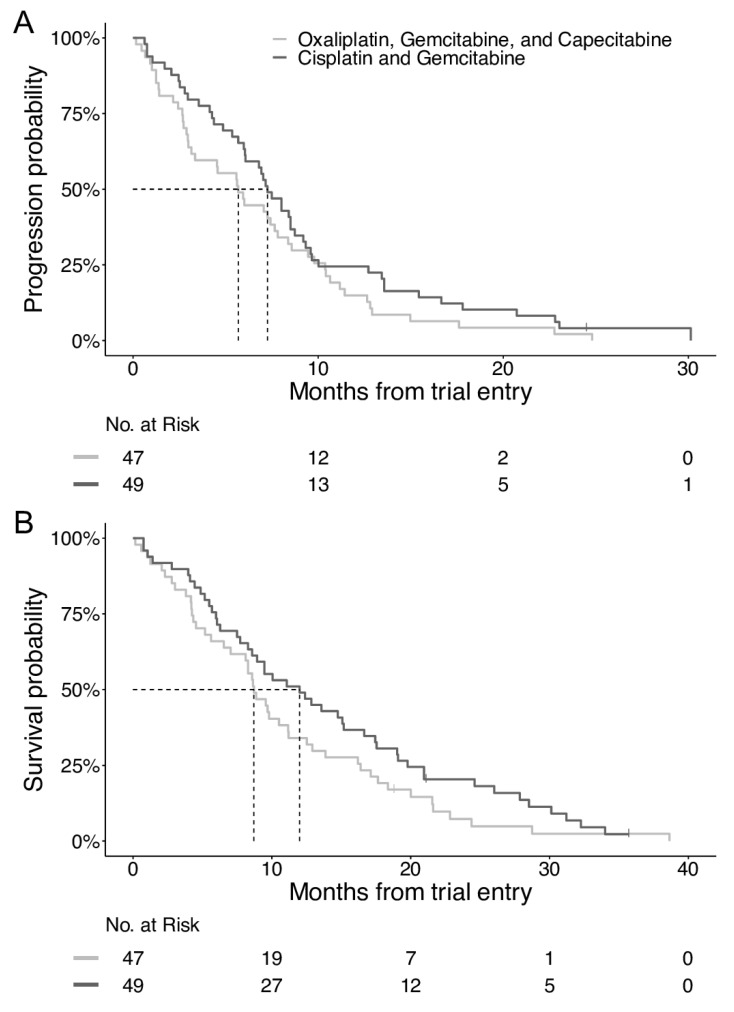
Kaplan–Meier plots of the progression-free survival (**A**) and overall survival (**B**).

The database was closed for analysis in August 2019. One-hundred patients were randomized, with 96 starting treatment: 47 with oxaliplatin, gemcitabine, and capecitabine and 49 with cisplatin and gemcitabine (Figure 1). At the time of the analysis, 93 patients had died, and 95 patients had either had progression of the disease or had died. The baseline characteristics were well-balanced between the two groups (Table 1), except for a statistically insignificant difference between patients with metastatic disease, with 57% in the oxaliplatin group versus 73% in the cisplatin group. The median PFS (mPFS) was 7.3 months (95% CI 6.0–8.7) in the cisplatin group and 5.7 months (95% CI 3.0–7.8) in the oxaliplatin group (HR = 0.721, *p* = 0.1) (Figure 2A). The mOS was 12.0 months (95% CI 8.3–16.7) and 8.7 months (95% CI 6.5–11.2), respectively (HR = 0.731, *p* = 0.1) (Figure 2B).

Tumor control (CR, PR, or SD) was achieved in 28 of the 47 patients (60%) in the oxaliplatin group and 39 of the 49 patients in the cisplatin group (79%) (*p* = 0.045) (Table 2). While no patients received a complete response, one patient in the cisplatin group received a response allowing radical surgery but had a relapse of the disease after four months.

Both regimes were well-tolerated, with few adverse events, but the cisplatin group had significantly more hematological toxicity (Table 3). As there was a significant difference in the hematological toxicity, we decided to investigate the doses delivered. We looked at cisplatin and oxaliplatin, as they are the drugs most likely to be reduced. It was carried out to clarify the degree of dose reductions as an indication that the dose could be too low. As this investigation was not preplanned, the intended dose was calculated based on the body surface area at the beginning of treatment and did not consider any dose reductions based on weight loss during treatment. Patients were censored at discontinuation of treatment or after 24 weeks.

**Figure 3:**
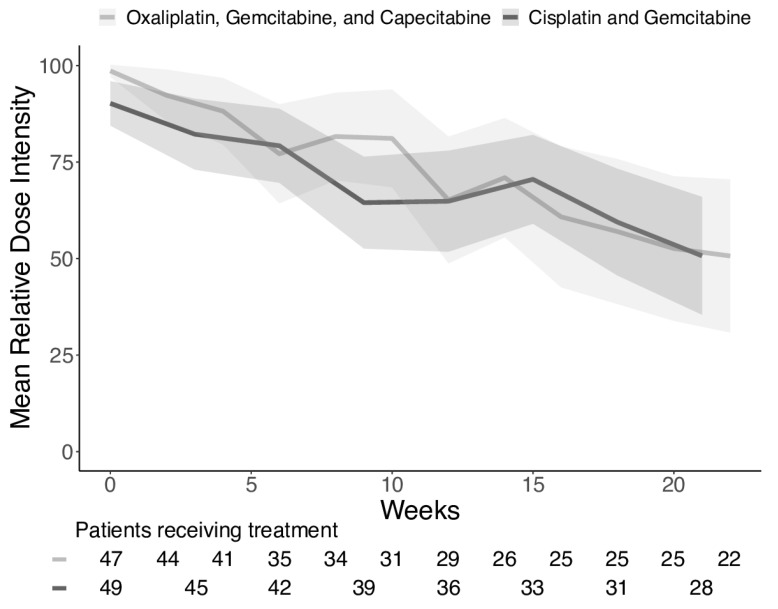
Mean relative dose intensity of the two treatments with 95% confidence intervals. It can be seen from the graph and the number of patients receiving treatment that both arms have similar patterns in both the dose reduction and the number of evaluable patients still receiving treatment as time progressed.

By exploring the mean relative DI, we found that both oxaliplatin and cisplatin were reduced to approximately 50% during treatment, either due to treatment delays or dose reductions (Figure 3). Only 22 patients (47%) received all 12 cycles of oxaliplatin, and only 6 patients (13%) received the full dose in the last cycle. The same was seen for cisplatin, with 28 patients (57%) receiving all eight cycles and seven patients (14%) receiving the full dose in the last cycle.

## 4. Discussion

This is the first randomized trial between treatment with oxaliplatin, gemcitabine, and capecitabine and treatment with cisplatin and gemcitabine for advanced BTC. The study was justified by preliminary data pointing toward a comparable effect but with less toxicity and a shorter infusion time with oxaliplatin, gemcitabine, and capecitabine. As a randomized phase II trial, formal statistical comparisons between the two treatments was not possible. However, we found it interesting, for an initial assessment of the two regimes based on outcomes, tolerability, and feasibility, as to whether a randomized phase III noninferiority trial in an international collaboration would be feasible.

In a systematic review comparing data from treatments with cisplatin and gemcitabine to oxaliplatin and gemcitabine in the first-line treatment of BTC, researchers found a similar weighted median of mOS of 9.7 months with cisplatin and 9.5 months with oxaliplatin [8]. As doses in the cisplatin group were heterogeneous, ranging from 25 mg/m^2^ at days 1 and 8 in a three-week schedule to 80 mg/m^2^ at day 1 in a three-week schedule, they looked at the six trials with cisplatin on days 1 and 8. The weighted median of the mOS increased from 9.7 to 11.7 months in these six trials with a lower dose intensity of cisplatin.

In trials with triple chemotherapy using oxaliplatin, gemcitabine, and capecitabine, lower doses of oxaliplatin were used, either 50–60 mg/m^2^ every second week in three trials [14,15,16] or 100 mg/m^2^ every third week [13]. The four trials [13,14,15,16] all had a similar mOS to the ABC-02 trial with cisplatin and gemcitabine.

Despite a mOS around 12 months from the four trials with oxaliplatin, gemcitabine, and capecitabine, we only found a mOS of 8.7 months in this trial. Although we used a lower dose of oxaliplatin in the experimental arm with oxaliplatin, gemcitabine, and capecitabine to avoid neutropenia, the oxaliplatin dose was still reduced to 50% during six months of treatment, and we believe that a higher initial dose of oxaliplatin would just lead to an increased dose reduction during the treatment period. Further, our results in the oxaliplatin group with a mOS of 8.7 months are comparable to the systematic review where a mOS of 9.5 months in the oxaliplatin group was found, even though higher doses of oxaliplatin were used.

In this trial, a mOS of 12 months with cisplatin and gemcitabine was found, despite a similar dose reduction of cisplatin during the six months. This mOS is comparable to the ABC-02 trial and the abovementioned review with cisplatin on days 1 and 8.

The purpose of this trial was to describe the effects and toxicity in patients with unresectable BTC treated with either oxaliplatin, gemcitabine, and capecitabine or cisplatin and gemcitabine. We observed a markedly higher mPFS of 7.3 months with cisplatin and gemcitabine compared to 5.7 months with oxaliplatin, gemcitabine, and capecitabine (HR = 0.731); however, this was not statistically significant (*p* = 0.1). Likewise, the mOS and tumor control was superior with cisplatin and gemcitabine. Both regimes were well-tolerated, with few nonhematological adverse events. As a preliminary investigation into the relevance and ethical feasibility of conducting a phase III noninferiority trial, we do not find the results encouraging. Although the study was not powered for a formal statistical comparison, we find that a difference of three months in the mOS is relevant and will not continue this trial in a phase III noninferiority setup.

## 5. Conclusions

Our conclusion is that, even though the triple combination was more convenient in terms of the infusion time and number of visits, cisplatin and gemcitabine should still be considered the standard first-line treatment for BTC.

## Figures and Tables

**Table 1:** Baseline characteristics of the study participants, according to the treatment group.

	Oxaliplatin Gemcitabine Capecitabine (*N* = 47)	Cisplatin Gemcitabine (*N* = 49)	Total *N* = 96	*p*-Value (Fisher’s Exact Test)
**Sex**				
Male Female	23 24	23 26	46 50	*p* = 1.0
**Age—years**				
Median (Range)	65 (21–85)	65 (39–82)	-	*p* = 0.87
**Performance status**				
0 1	23 24	23 26	46 50	*p* = 1.0
**Primary tumor site**				
Intrahepatic Extrahepatic Klatskin Gallbladder Unknown	23 4 5 14 1	31 1 4 11 2	54 5 9 25 3	*p* = 0.46
**Stage**				
Locally advanced Metastatic Unknown	20 (43%) 27 (57%) 0	12 (24%) 36 (73%) 1 (2%)	32 63 1	*p* = 0.08
**Earlier therapy**				
Curative surgery Adjuvant chemotherapy	7 7	3 2	10 9	N/A

**Table 2:** Best tumor response in the two treatment arms evaluated by the Response Evaluation Criteria in Solid Tumors (RECIST) 1.1. Tumor control was achieved in 28 of the 47 patients (60%) in the oxaliplatin group and 39 of the 49 patients in the cisplatin group (79%).

Treatment	Oxaliplatin, Gemcitabine and Capecitabine (*N* = 47)	Cisplatin and Gemcitabine (*N* = 49)
Complete response	0	0
Partial response	8 (17%)	8 (16%)
Stable disease	20 (43%)	31 (63%)
Progression	14 (30%)	5 (10%)
Not assessable	5 (11%)	5 (10%)

**Table 3:** Adverse events in the two treatment arms reported as any grade and subdivided as grades 3 and 4. In order to identify statistically significant differences in the toxicity, a Fisher’s exact test was performed on grade 3 and 4 toxicities. Statistically significant values are highlighted in bold.

Adverse Event	Oxaliplatin, Gemcitabine, and Capecitabine (*N* = 47)		Cisplatin and Gemcitabine (*N* = 49)		*p*-Value Grades 3+4 (Fisher’s Exact Test)
**Hematological**	**Any Grade** *N* (%)	**Grades 3–4** *N* (%)	**Any Grade** *N* (%)	**Grades 3+4** *N* (%)	
Anemia	10 (22%)	3 (7%)	17 (35%)	7 (14%)	*p* = 0.32
Neutropenia	4 (8%)	0 (0%)	30 (61%)	21 (43%)	***p* < 0.001**
Febrile neutropenia	0 (0%)	0 (0%)	5 (10%)	5 (10%)	*p* = 0.06
Thrombocytopenia	10 (22%)	1 (2%)	16 (33%)	12 (24%)	***p* = 0.002**
**Nonhematological**					
Fatigue	37 (80%)	1 (2%)	36 (73%)	3 (6%)	*p* = 0.62
Nausea/anorexia	35 (76%)	2 (4%)	34 (69%)	0 (0%)	*p* = 0.24
Vomiting	20 (43%)	2 (4%)	15 (31%)	1 (2%)	*p* = 0.61
Diarrhea	19 (41%)	2 (4%)	9 (18%)	1 (2%)	*p* = 0.61
Sensory neuropathy	41 (89%)	2 (4%)	24 (49%)	2 (4%)	*p* = 1.0
Tinnitus/hearing loss	1 (2%)	0 (0%)	10 (20%)	2 (4%)	*p* = 0.50
Hand–foot syndrome	20 (43%)	1 (2%)	0 (0%)	0 (0%)	*p* = 0.49
Biliary tract obstruction	9 (20%)	9 (20%)	2 (4%)	1 (2%)	***p* = 0.007**
Infection/fever	33 (72%)	11 (24%)	40 (82%)	10 (20%)	*p* = 0.81
Thromboembolic event	7 (15%)	3 (7%)	12 (24%)	10 (20%)	*p* = 0.07
